# Genome-wide compound heterozygote analysis highlights alleles associated with adult height in Europeans

**DOI:** 10.1007/s00439-017-1842-3

**Published:** 2017-09-18

**Authors:** Kaiyin Zhong, Gu Zhu, Xiaoxi Jing, A. Emile J. Hendriks, Sten L. S. Drop, M. Arfan Ikram, Scott Gordon, Changqing Zeng, Andre G. Uitterlinden, Nicholas G. Martin, Fan Liu, Manfred Kayser

**Affiliations:** 1 Department of Genetic Identification, Erasmus MC University Medical Center Rotterdam, Rotterdam, The Netherlands; 2 Queensland Institute of Medical Research, Brisbane, 4029 Australia; 3 Key Laboratory of Genomic and Precision Medicine, Beijing Institute of Genomics, Chinese Academy of Sciences, Beijing, China; 4 University of Chinese Academy of Sciences, Beijing, China; 5 Division of Endocrinology, Department of Pediatrics, Sophia Children’s Hospital, Erasmus MC University Medical Center Rotterdam, Rotterdam, The Netherlands; 6 Department of Pediatrics, University of Cambridge, Cambridge, UK; 7 Department of Internal Medicine, Erasmus MC University Medical Center Rotterdam, Rotterdam, The Netherlands; 8 Department of Epidemiology, Erasmus MC University Medical Center Rotterdam, Rotterdam, The Netherlands

## Abstract

Adult height is the most widely genetically studied common trait in humans; however, the trait variance explainable by currently known height-associated single nucleotide polymorphisms (SNPs) identified from the previous genome-wide association studies (GWAS) is yet far from complete given the high heritability of this complex trait. To exam if compound heterozygotes (CH) may explain extra height variance, we conducted a genome-wide analysis to screen for CH in association with adult height in 10,631 Dutch Europeans enriched with extremely tall people, using our recently developed method implemented in the software package CollapsABEL. The analysis identified six regions (3q23, 5q35.1, 6p21.31, 6p21.33, 7q21.2, and 9p24.3), where multiple pairs of SNPs as CH showed genome-wide significant association with height (*P* < 1.67 × 10^−10^). Of those, 9p24.3 represents a novel region influencing adult height, whereas the others have been highlighted in the previous GWAS on height based on analysis of individual SNPs. A replication analysis in 4080 Australians of European ancestry confirmed the significant CH-like association at 9p24.3 (*P* < 0.05). Together, the collapsed genotypes at these six loci explained 2.51% of the height variance (after adjusting for sex and age), compared with 3.23% explained by the 14 top-associated SNPs at 14 loci identified by traditional GWAS in the same data set (*P* < 5 × 10^−8^). Overall, our study empirically demonstrates that CH plays an important role in adult height and may explain a proportion of its “missing heritability”. Moreover, our findings raise promising expectations for other highly polygenic complex traits to explain missing heritability identifiable through CH-like associations.

**Electronic supplementary material:**

The online version of this article (doi:10.1007/s00439-017-1842-3) contains supplementary material, which is available to authorized users.

## Introduction

Human adult height is highly influenced by genetic factors with an estimated heritability of up to 80% (Carmichael and McGue 1995; Nielen et al. 2001; Silventoinen et al. 2003, 2008). A number of large-scale GWASs (Estrada et al. 2009; Liu et al. 2014; Wood et al. 2014; Yang et al. 2010), carried out to explore the genetic architecture underlying the variation in height, have successfully identified at close to 700 common single nucleotide polymorphisms (SNPs) (Wood et al. 2014), and more recently 83 low-frequency (Marouli et al. 2017) variants, showing genome-wide significant association with height. However, these SNPs together can only explain about 21.6% (Marouli et al. 2017) of the phenotypic variance in the study populations (combined sample size 711,428 individuals) due to the extremely small effect sizes for most associated SNPs. Large fractions of the “missing heritability” are also known to occur in a wide range of human complex traits and common disorders (Keller et al. 2012; Ridge et al. 2013; Tomlinson et al. 2011). Missing heritability represents the central challenge in understanding the genetics of common traits in humans, with consequences for future applications in different fields such as precision medicine and forensic genetics.

Aside from epigenetic factors, there are two mutually compatible leading theories for the major sources of this missing heritability. The first theory emphasizes that there may exist a large body of rare or low-frequency DNA variants, each with moderate-to-large effects (Bodmer and Bonilla 2008). Such variants are not detectable due to low statistical power, low array coverage, and low imputation quality for rare variants (Yang et al. 2015). Consequently, the key to solving the “missing heritability” puzzle is to scale up sample sizes together with genome-sequencing-based techniques, which are both time-consuming and expensive. For example, a very recent exome chip study of height carried out by the GIANT Consortium identified 32 extremely rare (MAF <0.01) height-associated variants, some with much larger effects than previously seen for common SNPs, using a data set consisting of 840,552 individuals (Marouli et al. 2017). The second theory stresses that variation in most quantitative phenotypes must result at least in part from multifactorial genetic perturbation of highly dynamic, interconnected biochemical networks (Mackay 2014), while the traditional association studies focus on additive effects of individual genes (Carlborg and Haley 2004; Manolio et al. 2009). The challenge here is that the abundance of complex forms of genetic interactions will be hard to detect due to the problem of combinatorial complexity that demands large-scale computational power.

Compound heterozygotes (CH) in classical genetics refer to two different mutations at a particular gene, one on each chromosome—both typically rare and deleterious (Schaaf et al. 2011), together cause an autosomal recessive trait. CH has been found in nearly all autosomal recessive disorders, where multiple mutations (i.e., different alleles) can affect the function of the gene product and lead to disease (Bezzina et al. 2003; Krude et al. 1998; Liu et al. 2011; Whittock et al. 2002), and therefore, a common genetic mechanism is assumed. Considering that trait-associated DNA variants identified from GWAS are typically non-coding due to the design of the SNP microarrays used, and can thus be involved in the regulation of gene expression (Visser et al. 2012, 2014, 2015), it is reasonable to expect that interactions involving non-coding DNA variants can also be highly relevant in the genetics of complex traits. Therefore, we recently proposed relaxed forms of CH, in which genetic variants physically close to one another [and in linkage disequilibrium (LD) to one another] are likely involved in a wide range of human polygenic traits and may present an important source of the “missing heritability” (Liu et al. 2016; Zhong et al. 2016a). Accordingly, we had developed a generalized collapsed double heterozygosity (GCDH) test to screen for CH-like association in GWASs. This method is implemented in the publically available R package CollapsABEL (Zhong et al. 2016a). Recently, we demonstrated that this approach was able to detect new genetic loci associated with the skin condition actinic keratosis via genome-wide GCDH analysis (Zhong et al. 2016b).

In the current study, we investigate if CH-like SNP interactions are associated with adult height in Europeans, and if so, to which extent they contribute to explaining the “missing heritability” previously noted for height with the conventional GWAS approach. For this, we used CollapsABEL to carry out genome-wide GCDH analysis to previously established genome-wide SNP array data of 10,361 Dutch Europeans from the Rotterdam Study (RS), including 770 additional Dutch Europeans of extremely tall stature (Liu et al. 2014), and used data from 4080 unrelated individuals of European origin from the Queensland Institute of Medical Research (QIMR) as a replication data set.

## Results

**Fig. 1:**
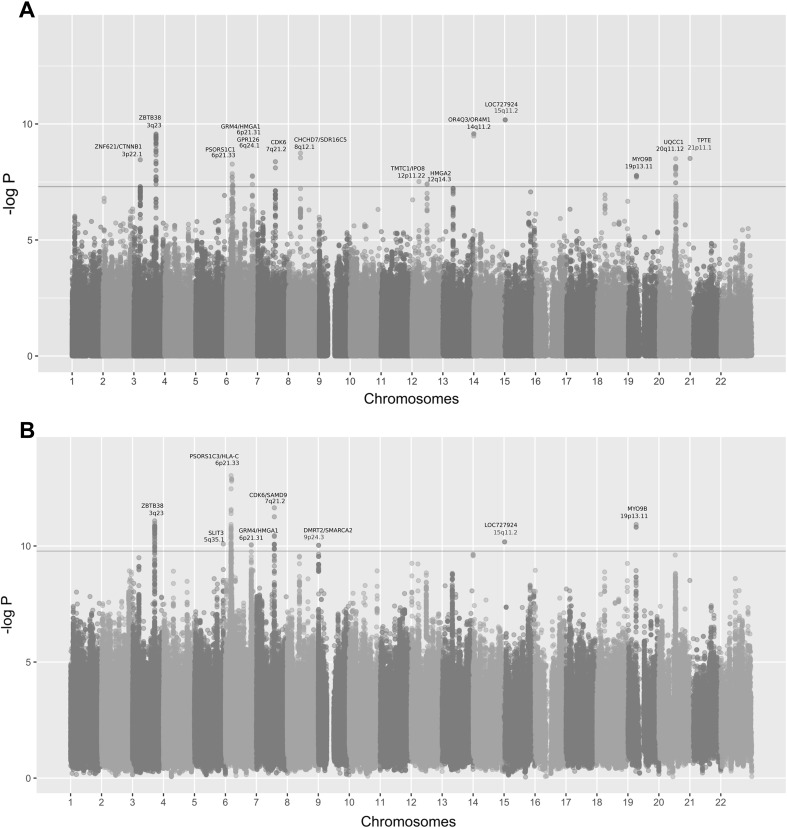
Manhattan plots from RS. Minus log of *P* value is represented by *blue* and *red dots* and grouped by chromosome. **a** Manhattan plot for single-SNP GWAS. The *horizontal line* (−log 5 × 10^−8^) indicates threshold of genome-wide significance. **b** Manhattan plot for GCDH scan, the *horizontal line* (−log 1.67 × 10^−10^) indicates threshold of GCDH genome-wide significance. Loci are annotated in *green* if they have not been reported before with genome-wide height association by the GIANT consortium (Wood et al. 2014)

**Fig. 2:**
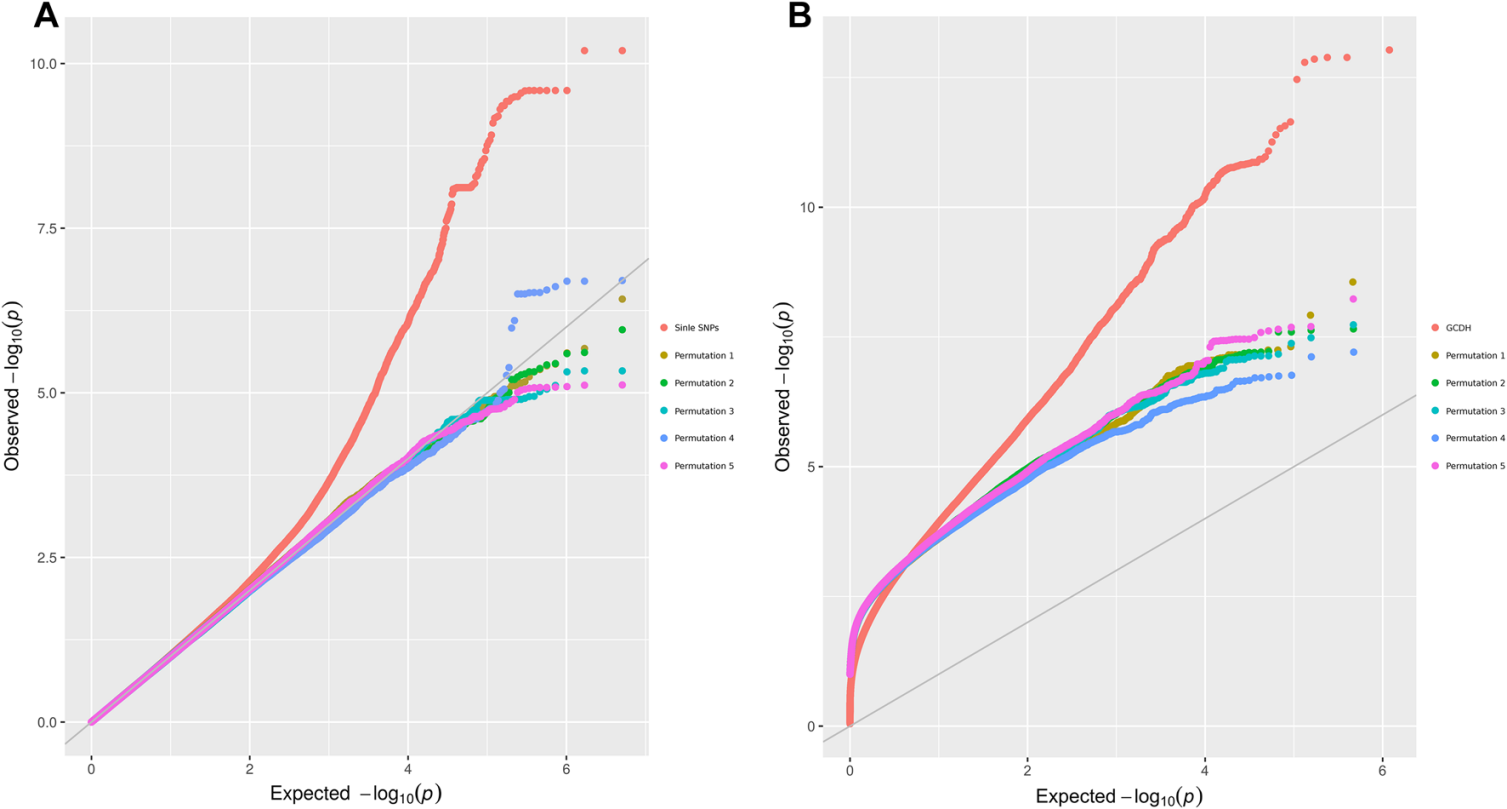
QQ plots. **a** QQ plot of single-SNP *P* values. **b** QQ plot of GCDH *P* values. In both cases, *P* values from five permutation analyses were also plotted to estimate the null distribution

Characteristics of the study populations are listed in Online Resource 1. A conventional GWAS in 10,361 Dutch Europeans from the RS including 770 extremely tall Dutch Europeans identified 101 SNPs from 14 loci (Fig. 1a and Online Resource 2), showing genome-wide significant association with height (*P* < 5 × 10^−8^). The top-associated SNPs at each locus were rs12485899 from 3p22.1 (*ZNF621*, *P* = 3.50 × 10^−9^, *β* = −0.57), rs9838625 from 3q23 (*ZBTB38*, *P* = 2.71 × 10^−10^, *β* = 0.62), rs1265097 from 6p21.33 (*PSORS1C1*, *P* = 5.39 × 10^−9^, *β* = −0.89), rs2780226 from 6p21.31 (*GRM4/HMGA1*, *P* = 4.26 × 10^−8^, *β* = 0.92), rs7741741 from 6q24.1 (*GPR126*, *P* = 1.72 × 10^−8^, *β* = −0.6), rs4272 from 7q21.2 (*CDK6*, *P* = 4.23 × 10^−9^, *β* = 0.68), rs6984782 from 8q12.1 (*CHCHD7*, *P* = 1.80 × 10^−9^, *β* = −0.94), rs4931222 from 12p11.22 (*TMTC1*, *P* = 3.05 × 10^−8^, *β* = −1.36), rs1038196 from 12q14.3 (*HMGA2*, *P* = 3.86 × 10^−8^, *β* = −0.53), rs7159961 from 14q11.2 (*OR4Q3*, *P* = 2.68 × 10^−10^, *β* = −2.66), rs1366870 from 15q11.2 (*LOC727924*, *P* = 6.67 × 10^−11^, *β* = −2.34), rs2279007 from 19p13.11 (*MYO9B*, *P* = 1.65 × 10^−8^, *β* = −0.65), rs6060355 from 20q11.22 (*UQCC1*, *P* = 3.15 × 10^−9^, *β* = 0.59), and rs10439884 from 21p11.1 (*TPTE*, *P* = 3.06 × 10^−9^, *β* = −2.16). Among these, two loci (15q11.2 and 21p11.1) represent novel findings, but at both loci, the top-associated SNPs had relatively low minor allele frequency (MAF, at 1–2%), while the remaining 12 loci have been previously identified by the GIANT Consortium via traditional GWAS (Wood et al. 2014). Note that the test statistics from our conventional GWAS slightly deviated from the expected distribution under the null hypothesis of no association (*λ* = 1.05, Fig. 2a). In contrast, the test statistics from a permutation analysis (*k* = 5, Fig. 2a) followed the expected distribution under the null tightly, suggesting that the significant signals are unlikely false positives due to residual population substructures.

We then conducted an exact replication analysis of these 14 SNPs in 4080 unrelated Australian Europeans from the Queensland Institute for Medical Research (QIMR). The two novel loci which had very low MAF in QIMR (0.1–0.2%) are thus not available for replication. For the remaining 12 SNPs, 10 showed the same allele effect direction as in the RS and 6 showed nominally significant association with height (*P* < 0.05, Online Resource 2), highlighting *GRM4/HMGA1*, *GPR126*, *CDK6*, *HMGA2*, *MYO9B*, and *UQCC1* genes. The replication rate of the single SNPs was lower than expected from our power analysis, which showed a >90% probability of replicating all the 14 SNPs (see Online Resource 3). The lower replication rate may be explained by multiple factors. For example, allelic heterogeneity has been previously noted as a frequent feature underlying the genetic architecture of adult (Allen et al. 2010). Notably, the novel loci 15q11.2 and 21p11.1 did not replicate because of the very low MAF (0.1–0.2%) in QIMR.

**Fig. 3:**
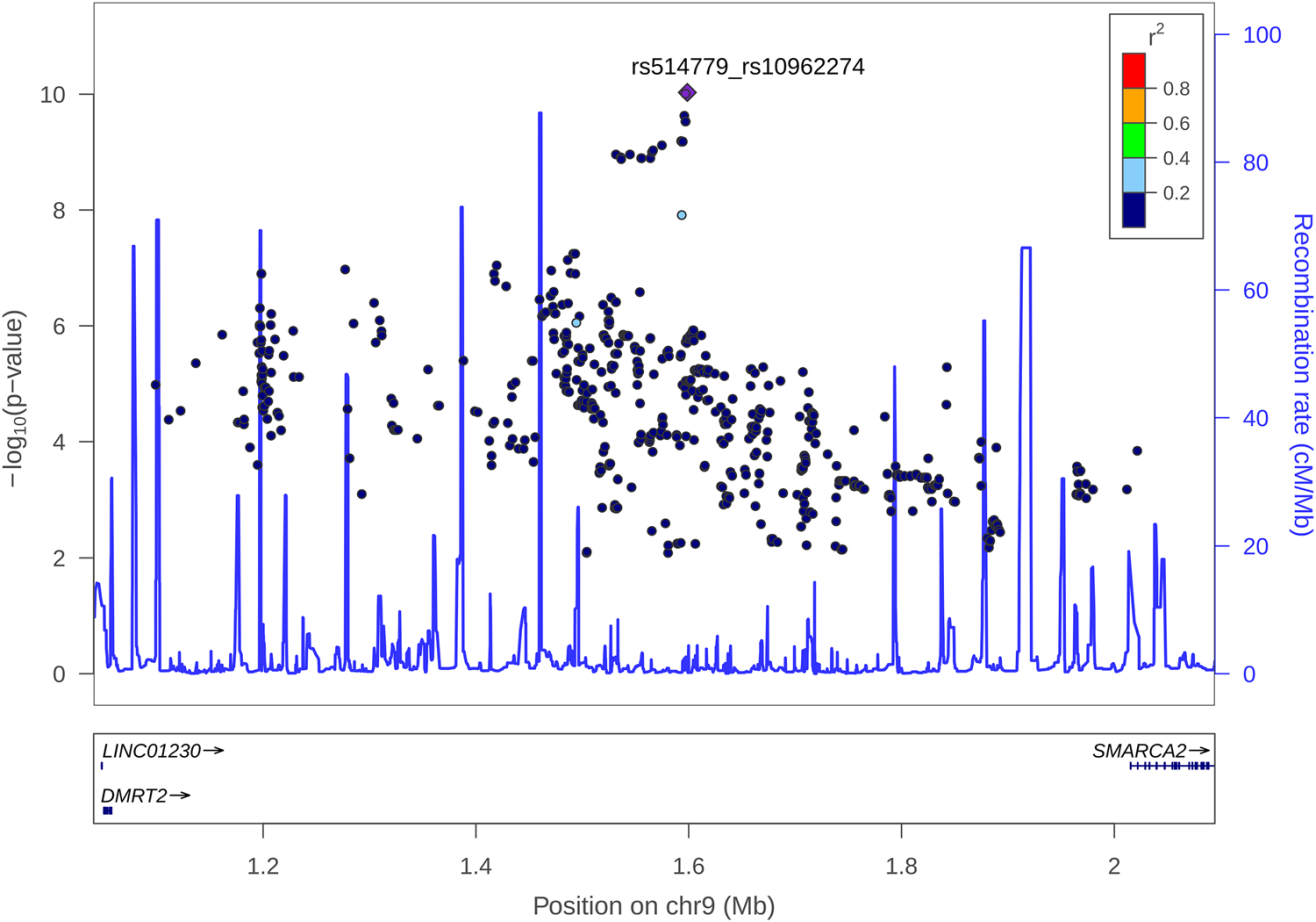
Regional Manhattan plot of GCDH *P* values from 9p24.3

We conducted a genome-wide screening for relaxed forms of compound or double heterozygotes that are potentially associated with adult height in the RS. This analysis was conducted using our recently developed software package CollapsABEL implementing a generalized collapsed double heterozygosity test (GCDH). The GCDH employs a generalized linear modeling framework to test the association between a pseudo-marker and a phenotype with or without covariates, where the pseudo-marker is collapsed from two physically close SNPs, as described in more details in the “Materials and methods”. The test statistics from the GCDH genome screening (Fig. 2b) showed a large deviation (*λ* = 4.16) from the expected null of the conventional GWAS as the result of multiple testing from the sliding window approach implemented in CollapsABEL. This deviation is expected, because first, CollapsABEL only reports the most significant association signal per window, i.e., a problem of multiple testing of correlated SNPs, and second, a p-filter was applied to pre-remove SNPs with little or no marginal effect. Therefore, we conducted a permutation analysis to approximate the null empirically, i.e., repeating the full GCDH genome screening five times using permuted phenotype values, and considered the distribution of the resultant test statistics as an empirical null distribution (Fig. 2b). Although the number of permutation is small due to the computational burden, it is obvious that the GCDH results deviated substantially from the empirical null distribution, which is unlikely explained by false-positive findings (Fig. 2b). This GCDH screen provided genome-wide significant evidence for six genetic regions consisting of CH-like association signals from the collapsed pseudo-markers (3q23, 5q35.1, 6p21.31, 6p21.33, 7q21.2, and 9p24.3, *P* < 1.67 × 10^−10^, see Fig. 1b; Table 1 and Online Resource 4). Notably, none of the individual SNPs in these regions showed genome-wide significant association in our conventional GWAS (*P* > 5 × 10^−8^). The top-associated pseudo-markers were collapsed from SNP pairs in these six regions, including rs9821337_rs6762826 from 3q23 (*ZBTB38*, *P* = 9.59 × 10^−11^, *β* = 0.62), rs1466947_rs17070997 from 5q35.1 (*SLIT3*, P = 8.21 × 10^−11^, *β* = −0.78), rs1776897_rs2744977 from 6p21.31 (*GRM4/HMGA1/C6orf106*, *P* = 1.61 × 10^−13^, *β* = 0.78), rs6904669_rs1960278 from 6p21.33 (*PSORS1C3/HLA*-*C*, *P* = 9.32 × 10^−14^, *β* = −0.89), rs7793983_rs17164894 from 7q21.2 (*CDK6/SAMD9*, *P* = 3.56 × 10^−11^, *β* = 0.87), and rs514779_rs10962274 from 9p24.3 (*DMRT2/SMARCA2*, *P* = 9.34 × 10^−11^, *β* = −0.98). One of the six loci, 9p24.3 (see also the regional plot, as shown in Fig. 3), represents a novel finding, whereas the other five (see regional plots in Online Resource 5) had been previously described as influencing height variation by the GIANT Consortium from a large-scale conventional GWAS (Wood et al. 2014). Significant GCDH signals were detected at two additional loci, 15q11.2 and 19p13.11, but are potentially explained by strong single-SNP association effects rather than the effect of a pair of SNPs, since individual SNP analysis already detected those SNPs as genome-wide significant (rs1366870 and rs2217377 in 15q11.2 and rs11671774, rs2279008, and rs2279007 in 19p13.11, see Fig. 1).

**Table 1:** Discovery and replication of six CH SNP pairs

Chr	Genes	SNP_1_	EA_1_	fEA_1_	SNP_2_	EA_2_	fEA_2_	*P*_1_	*P*_2_	*P*	*P*_rep_
3q23	ZBTB38	rs9821337	G	0.38	rs6762826	G	0.09	1.69 × 10^−7^	2.77 × 10^−2^	9.59 × 10^−11^	**1.49** **×** **10** ^**−8**^
5q35.1	SLIT3	rs1466947	G	0.11	rs17070997	T	0.07	1.21 × 10^−7^	1.13 × 10^−4^	8.21 × 10^−11^	8.62 × 10^−1^
6p21.33	PSORS1C3/HLA-C	rs6904669	G	0.41	rs1960278	C	0.43	3.62 × 10^−1^	3.09 × 10^−6^	9.32 × 10^−14^	1.75 × 10^−1^
6p21.31	GRM4/HMGA1/C6orf106	rs1776897	G	0.09	rs2744977	G	0.16	5.32 × 10^−8^	8.17 × 10^−8^	1.61 × 10^−13^	**9.30** **×** **10** ^**−3**^
7q21.2	CDK6/SAMD9	rs7793983	G	0.09	rs17164894	A	0.07	2.30 × 10^−6^	1.65 × 10^−5^	3.56 × 10^−11^	5.27 × 10^−1^
9p24.3	DMRT2/SMARCA2	rs514779	C	0.03	rs10962274	C	0.08	1.51 × 10^−5^	1.98 × 10^−4^	9.34 × 10^−11^	**2.58** **×** **10** ^**−2**^

*EA*_*1*_ *, EA*_*2*_ effect alleles for the first and second SNPs in RS data set, *fEA*_*1*_ *, fEA*_*2*_ minor allele frequency for the first and second SNPs in RS data set, *P*_*1*_ *, P*_*2*_ single-SNP *P* value for the first and second SNPs in RS data set, *P*_GCDH_ *P* value in RS data set, and *P*_*rep*_ *P* value from replication analysis in QIMR data set, where* P* values < 0.05 are highlighted with bold font

We then conducted an exact replication analysis of these six SNP pairs in the QIMR data set. The newly identified locus 9p24.3 (rs514779_rs10962274, *P* = 0.026, *β* = −0.48) as well as three previously known markers were replicated at nominal significance, i.e., rs9821337_rs6762826 from 3q23 (*P* = 1.49 × 10^−8^, *β* = 0.72), rs1776897_rs2744977 from 6p21.31 (*P* = 0.0093, *β* = 0.37), rs17688839_rs17164894 from 7q21.2 (*P* = 0.0019, *β* = 0.44). All of these replicated *β* values showed the same directions as those in the Dutch Europeans (Table 1).

A previous large-scale meta-analysis of height GWASs identified 697 SNPs at different loci independently contributing to the variation in adult height and 658 SNPs were available in our RS data set. The proportion of sex- and age-adjusted height variance explainable by these 658 SNPs in our data set was estimated at 19.13% by constructing a polygenic risk score based on the weighted allele sums of all 658 SNPs (Table 2). This polygenic score is referred to below as PRS658. A multivariable analysis further demonstrated that on top of the PRS658, the six top-associated pseudo-markers collapsed from six pairs of SNPs explained 2.51%, and the 14 top-associated SNPs from our conventional GWAS explained 3.23% of the sex- and age-adjusted height variance (Table 2). For all six loci, the top-associated pseudo-marker explained a substantially higher proportion of the trait variance than individual SNPs of a pair, i.e., when any of the two SNPs was tested in a univariate model (Online Resource 6), and explained a higher or similar proportion of the trait variance than the two SNPs together, i.e., when both SNPs were included in a multiple regression model (Online Resource 6). This indicates that including the pseudo-markers in prediction modeling may not necessarily boost the prediction accuracy compared with the models including all pairs of SNPs in CH. However, it should be emphasized that the use of the pseudo-marker provides a substantially higher power in detecting CH-like association signals than the conventional GWAS.

**Table 2:** Multivariable linear model including 14 single SNPs, 6 CH SNP pairs and the weighted allele sum of 658 SNPs previously reported to be associated with height as independent variables

Variable	Cytoband	Gene	−log_10_ *P*	*R* ^2^
PRS658			945.12	19.13
rs10439884	21p11.1	TPTE	19.22	0.32
rs7159961	14q11.2	OR4Q3	17.25	0.33
rs1366870	15q11.2	LOC727924	16.33	0.31
rs2279007	19p13.11	MYO9B	15.53	0.44
rs4931222	12p11.22	TMTC1	13.10	0.29
rs12485899	3p22.1	ZNF621	11.35	0.30
rs4272	7q21.2	CDK6	4.99	0.28
rs6984782	8q12.1	CHCHD7	3.33	0.26
rs7741741	6q24.1	GPR126	3.25	0.30
rs1038196	12q14.3	HMGA2	2.10	0.23
rs9838625	3q23	ZBTB38	1.93	0.12
rs1265097	6p21.33	PSORS1C1	1.89	0.04
rs6060355	20q11.22	UQCC1	1.38	0.01
rs2780226	6p21.31	GRM4	0.90	0.01
rs514779_rs10962274	9p24.3	DMRT2/SMARCA2	19.20	0.39
rs1466947_rs17070997	5q35.1	SLIT3	13.17	0.39
rs7793983_rs17164894	7q21.2	LOC101927497	11.65	0.50
rs6904669_rs1960278	6p21.33	PSORS1C3/HLA-C	10.39	0.48
rs1776897_rs2744977	6p21.31	GRM4/HMGA1	4.02	0.39
rs9821337_rs6762826	3q23	ZBTB38	0.76	0.36
Total 1^a^				2.51
Total 2^b^				3.23
Total 3^c^				24.87

^a^Total percentage of variation in height explained by 6 CH SNPs

^b^Total percentage of variation in height explained by 14 single SNPs

^c^Total percentage of variation in height explained by all independent variables in the model

Diplotype configuration inference supports the involvement of compound heterozygotes for three of the six pseudo-markers (3q23, 6p21.33, and 7q21.2) and the involvement of double (non-compound) heterozygotes (see Online Resource 7 for an illustration and double and compound heterozygosity) for the other three pseudo-markers (Online Resource 8).

## Discussion

An empirical application of the GCDH approach (as implemented in CollapsABEL) to height identified novel CH-like interactions and markers that are likely not identifiable by the conventional GWAS. With only a small fraction of the sample size of the GIANT study (Wood et al. 2014), we found CH-like interactions among known height-related SNPs and one previously unknown marker, which in turn explained a sizable percentage of variance in height. It is, therefore, expected that an application of CollapsABEL to the full GIANT data set (Wood et al. 2014) will reveal many more CH-like interactions which may include novel height loci not identifiable via standard single-SNP GWAS.

The two SNPs rs514779 and rs1853417 from the novel region 9p24.3 are located between the genes *DMRT2* and *SMARCA2*. *DMRT2* is a candidate gene for sex determination and is also involved in development of somites and muscle tissue in the embryo (Ottolenghi et al. 2000; Ounap et al. 2004; Seo 2007). Both functions may be important in determining adult height, although *DMRT2* has not been reported to be associated with height in the literature. *SMARCA2* encodes a protein, that is, part of the ATP-dependent chromatin remodeling complex SNF/SWI, which is required for transcriptional activation of genes normally repressed by chromatin (Koga et al. 2009). The interaction between the transcription-regulator gene *SMARCA2* and the development-related gene *DMRT2* makes perfect sense, although it needs further functional study to be verified.

The other five loci highlighted via CH-like effects (3q23, 5q35.1, 6p21.31, 6p21.33, and 7q21.2) have all been previously identified via large-scale traditional single-SNP GWAS of the GIANT Consortium (Wood et al. 2014). However, the CH-like interactions of SNPs found here had not been reported before. These results indicate that CH-like interactions among known loci may explain a sizable proportion of the phenotypic variance. The six best CH SNP pairs chosen from each significant locus accounted for 2.51%, compared to 3.23% by the 14 single SNPs from RS and 19.13% by PRS658. This clearly demonstrates that phenotypic variance explained by genetic CH-like interactions can be orthogonal to that attributed to single SNPs, a finding for height that is expected for other complex traits as well.

Notably, we have used an additive model, i.e., the alternative collapsing matrix (Zhong et al. 2016a), for genotype collapsing, which does not strictly differentiate between compound heterozygosity and non-compound double heterozygosity or any form of pairwise interaction between two variants showing an additive effect. As a consequence, the SNP pairs discovered cannot be expected to be all in CH. According to our diplotype inference results (Online Resource 8), GCDH signals were confirmed in three (3q23, 6p21.33, and 7q21.2) of the six most significant pseudo-markers. This implies that the relaxed form of CH is an important mechanism among the possible interaction forms conforming to the additive model, although non-compound double heterozygosity and possibly other kinds of genetic interactions may also play a significant role. Besides the additive model, other possible models (collapsing matrices) representing more complex/unknown genetic mechanisms could be explored in the future, due to the design flexibility of CollapsABEL.

Compared to recent large-scale traditional single-SNP GWASs for height carried out by the GIANT Consortium, consisting of 253,288 (Wood et al. 2014) up to 381,625 (Marouli et al. 2017) samples, the current study is of relatively small sample size, which limits statistical power. In addition, the preprocessing of genotype data with a *P* value filter, which removes all SNPs without a strong enough marginal effect, helped reducing computational burden, but also constrained the scope of discoverable interactions. However, the main goal of our study was to find out if and to what extent can the signals detected by CollapsABEL explain “missing heritability”. To maximize statistical power in identifying novel CH variants, we included a set of 770 extremely tall individuals in the RS data set. It had been shown previously that the effects of height-associated common DNA variants are consistent with the predicted polygenic effects in extremely tall persons (Chan et al. 2011). This consistency is the basis for a successful replication in the QIMR data set which does not include extremely tall subjects. Our previous study (Liu et al. 2014) using the same RS data also confirmed that the genetics of tall stature is similar to that of normal height in humans, i.e., heritability is for a large part explained by many common variants with small effects and allelic heterogeneity is a frequent feature, where CH represents a common form of allelic heterogeneity.

In comparing the replication results from single-SNP and GCDH approaches, we found that pseudo-markers collapsed from pairs of SNPs had a higher replication rate than did individual SNPs (50% of the loci were replicated for pseudo-markers vs. 35% for individual SNPs). This can be explained by the much stricter genome-wide significance threshold used in GCDH, i.e., the fewer false positives lead to higher reproducibility.

Although our method provides a higher power to detect CH-like associations, it cannot pinpoint causal variants as does in the conventional GWAS, particularly when the associated SNPs are intergenic. Therefore, our method cannot distinguish between two variants that affect the same or different genes and the finding at DMRT2 and SMARCA2 requires further functional studies.

Overall, the GCDH approach seems to be sitting at the saddle point of the two leading theories for “missing heritability”: it can detect CH-like interactions between pairs of SNPs often less frequent than what is detectable by the conventional GWAS. For future applications with larger sample sizes and adequate computing power in combination with whole genome-sequencing-based techniques, we expect GCDH to explain a considerably larger percentage of the variance, thus providing a partial explanation for “missing heritability” and new insights into genetics of human complex traits and diseases.

## Materials and methods

### The Rotterdam Study

The Rotterdam Study (RS) is a prospective population-based data set study of 14,926 participants’ aged 45 years and older, living in a suburb of Rotterdam, The Netherlands. Details of the study design and population have been described elsewhere (Hofman et al. 2015). The RS has been approved by the Medical Ethics Committee of the Erasmus MC and by the Ministry of Health, Welfare and Sport of The Netherlands, implementing the Wet Bevolkingsonderzoek: ERGO (Population Studies Act: Rotterdam Study). All participants provided written informed consent to participate in the study and to obtain information from their treating physicians. The RS was conducted according to the Declaration of Helsinki Principles.

Whole blood DNA extraction, genotyping, quality controls, and 1000 Genome (Consortium 2012)-based genotype imputation have been described in detail previously (Kreiner-Moller et al. 2015). In brief, genotyping was carried out using the Infinium II HumanHap550 BeadChips version 3 (Illumina, San Diego, California USA) and Human610-Quad BeadChips. After all quality controls, the current study included a total of 2,543,887 autosomal SNPs (MAF >0.01, imputation *R* ^2^ > 0.3, SNP call rate >0.95, and HWE >1 × 10^−3^) in 10,631 individuals. Height was measured using a stadiometer (SECA225; SECA, Hamburg, Germany). We also included 770 extremely tall subject from the Dutch Tall cohort (DT), which used the following inclusion criteria: (1) standard deviation score (SDs) above 1.88 according to Dutch standards (http://www.tno.nl/groei), which corresponds to the 3% upper tail of the height distribution in Dutch adults, approximately >195 cm in men and >180 cm in women at age 30, after correcting for secular trend, and (2) Dutch European ancestry defined as being born to Dutch parents who themselves were born in The Netherlands.

### The Queensland Institute of Medical Research (QIMR) study

The QIMR study comprised 8672 individuals from predominantly population and family-based studies, around 4161 from nuclear families living in or near Brisbane, Australia, with twins aged around 12–14 years at recruitment for clinical studies, and the remainder from older twin families across Australia recruited through the Australian Twin Registry. Genotyping was a subset of an existing larger data set which used Illumina SNP array chips in large batches from two chip families. Per-batch QC removed SNPs with any of (1) MAF <1%; (2) call rate <95%; (3) HWE *P* value <10^−6^; (4) mean GenCall score <0.7; (5) fail GenomeStudio filters as per Illumina QC SOPs; and (6) blacklists based on poor retest consistency and mapping issues, particularly gender-specific heterozygosity and call-rate filters for chromosome X. Genotyping of the 8672 was divided between first-generation Illumina chips (5960 of which 4300 were from 610 K-quad or (rarely) 660 K-quad chips; 1660 from 370 K or 317 K chips), and newer Omni and Core + Exome Illumina chips (2712 of which 2279 were from Core + Exome or PsychArray chips; 364 from Omni-2.5; 69 from Omni-Express). Imputation was to the 1000 Genomes Phase 3 mixed-population panel via the University of Michigan’s Imputation Server in March–April 2015, as one run per chromosome for each of the two chip families above, in each case for the observed markers available post-QC across all batches for that chip family (277,690 markers for first generation and 240,297 markers for Omni/Core + Exome); the two families merged post-imputation with a binary ‘run number’ covariate in the analysis to mitigate differences in imputation quality. The current study included 4080 unrelated QIMR subjects for replication of the associated SNPs.

### GCDH genome screening

We conducted a genome-wide GCDH screening of adult height for finding CH-like association in the RS using CollapsABEL. Important parameters of CollapsABEL include the collapsing matrix, the window size, and the p-filter. The collapsing matrix is constructed according to the underlying genetic model, and defines the way that the two SNPs under investigation are collapsed. Assuming a joint recessive model for the minor alleles of two nearby SNPs, one may choose the default collapsing matrix, based on which the two SNPs is collapsed into a pseudo-marker consisting of two possible pseudo-genotypes (0 or 1 minor allele vs. 2 or more minor alleles). Alternative collapsing matrices may be of choice when different genetic models are assumed. In the present application of height, we assumed a joint additive effect of the minor alleles of two nearby SNPs and thus used an alternative collapsing matrix, based on which the two SNPs under investigation are collapsed into a pseudo-marker consisting of three possible pseudo-genotypes (0, 1, and 2 or more minor alleles). The difference between compound and double heterozygotes is illustrated in Online Resource 7. It is important to note that compound and double heterozygotes are collapsed into the same pseudo-genotype and thus were not differentiated during the genome screening stage, but were differentiated in the subsequent analysis of the associated regions, where the haplotypes were inferred based on the expectation–maximization algorithm as implemented in the haplo.em function of the R package haplo.stats (Schaid et al. 2002). When results from haplo.stats show that the CH group has higher/lower mean height compared to the group with reference alleles in the same direction as *β* values from GCDH analysis, we consider it as a validation of the CH model. In the absence of CH group, we consider it an indication of involvement of other genetic mechanisms.

The genotype collapsing is done for all pairs of nearby SNPs within a sliding window which slides over the whole genome with one SNP per step. The size of the sliding window is defined by two criteria, the number of the maximal SNPs (*k*) and the maximal distance (*d*) between the index SNP and its pairing SNP. For each window, the first SNP is considered as the index SNP and all the remaining pairing SNPs satisfying both criteria are iteratively collapsed with the index SNP and all collapsed pseudo-markers are iteratively tested for association with the phenotype. Therefore, the maximal number of tests per window is *k*. The minimal *P* value within each window is assigned to the index SNP. In the current study of height, we used *k* = 300 and *d* = 500 kbp, and reported all pairs of SNPs with *P* values smaller than 1.67 × 10^−10^ (i.e., 5 × 10^−8^/300) from our GCDH genome screening, i.e., Bonferroni correction of the maximal possible number of tests per window.

The p-filter defines the inclusion criteria for SNPs with a minimal marginal effect in the conventional GWAS. In the current study, we set p-filter = 0.1 to achieve the balance between SNP coverage and the affordable computational burden.

The null distribution of the test statistics from CollapsABEL is expected to be highly inflated, because first, the program only reports the most significant association signal per window, i.e., a problem of multiple testing of correlated SNPs, and second, a p-filter was applied to pre-remove SNPs with little or no marginal effect. Since it was difficult to derive the null mathematically, we conducted a permutation analysis to approximate the null empirically. The whole GCDH genome screening procedure was performed five times using identical parameters, where the genotype–phenotype relationship was broken by randomly shuffling the order of individuals in the phenotype file (height, sex, age, and four PCs) and the resulting test statistics are displayed in QQ plots to illustrate the data-specific null distribution of our GCDH scan for adult height in the RS.

The conventional GWAS for adult height was conducted using PLINK v1.9 assuming an additive allele effect and considering sex, age, and four top genome PCs from the MDS analysis as covariates. Regional Manhattan plots were generated using Locuszoom (Pruim et al. 2010). Power analysis for the QIMR data set was conducted using the pwr R package (Champely 2013), assuming that *R* ^2^ is consistent across RS and QIMR for all the SNPs tested.

### Multivariable and replication analyses

A previous large-scale meta-analysis of height GWASs identified 697 SNPs at different loci independently contributing to the variation in adult height and 658 SNPs was available in our RS data set. We estimated the proportion of sex- and age-adjusted height variance explainable by these 658 SNPs in our data set by constructing a polygenic risk score based on the weighted allele sums of all 658 SNPs. This polygenic score is referred to below as PRS658. We then accessed the independent effects of PRS658, the SNPs showing genome-wide significant association with height in our conventional GWAS, and the pseudo-markers showing genome-wide significant association with height in our GCDH genome screen using a multivariable linear model. More specifically, this model considered sex- and age-regressed height residuals as the dependent variable and included PRS658, the 14 top-associated SNPs from our conventional GWAS, and the 6 top-associated pseudo-markers collapsed from six pairs of SNPs from the GCDH genome screen as the explanatory variables. The multivariable analysis was conducted in an iterative manner to access the *R* ^2^ change due to individual factors using R scripting, i.e., by adding one independent variable at a time to a linear model in ascending order according to the *P* values from the multivariable analysis including all independent variables; the *R* ^2^ estimated for the added independent variable then becomes the *R* ^2^ difference between the current model and a previous model. To further compare the height variance explained by the collapsed pseudo-markers and by the individual SNPs, we used sex- and age-adjusted residuals as the dependent variable and the collapsed pseudo-marker as the explanatory variable, which is compared with the model using the two individual SNPs of the pair as the explanatory variables.

We conducted a replication analysis for the 14 top-associated SNPs (one SNP per associated region) from the conventional GWAS and the six top-associated pseudo-markers (one per region) from the GCDH genome screen in 4080 unrelated QIMR subjects using linear regression adjusted for sex and age. *P* values smaller than 0.05 were considered as significant replication.

## Electronic supplementary material

Below is the link to the electronic supplementary material.

Supplementary material 1 (XLSX 9 kb)

Supplementary material 2 (XLSX 11 kb)

Supplementary material 3 (TIFF 57 kb)

Supplementary material 4 (XLSX 42 kb)

Supplementary material 5 (DOCX 2170 kb)

Supplementary material 6 (PDF 15 kb)

Supplementary material 7 (DOCX 844 kb)

Supplementary material 8 (XLSX 67 kb)
