# Supplementary figures and images for: Genome-wide compound heterozygote analysis highlights alleles associated with adult height in Europeans
Source: Hum Genet. 2017 Sep 18;136(11):1407–17. doi: 10.1007/s00439-017-1842-3 (PMC5702380; doi:10.1007/s00439-017-1842-3)

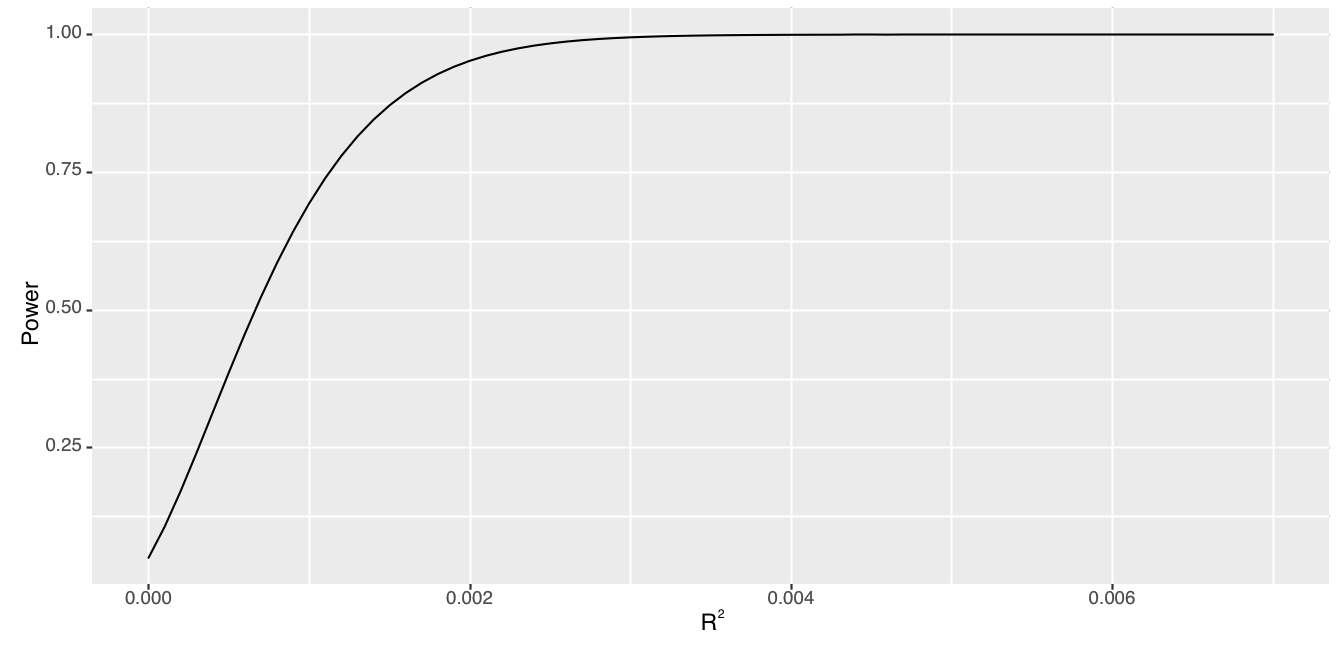

Supplement: Supplementary file 3 — Supplementary material 3 (TIFF 57 kb) [file 439_2017_1842_MOESM3_ESM.tiff]

Regional plots of 5 loci with significant CH signals in RS dataset.


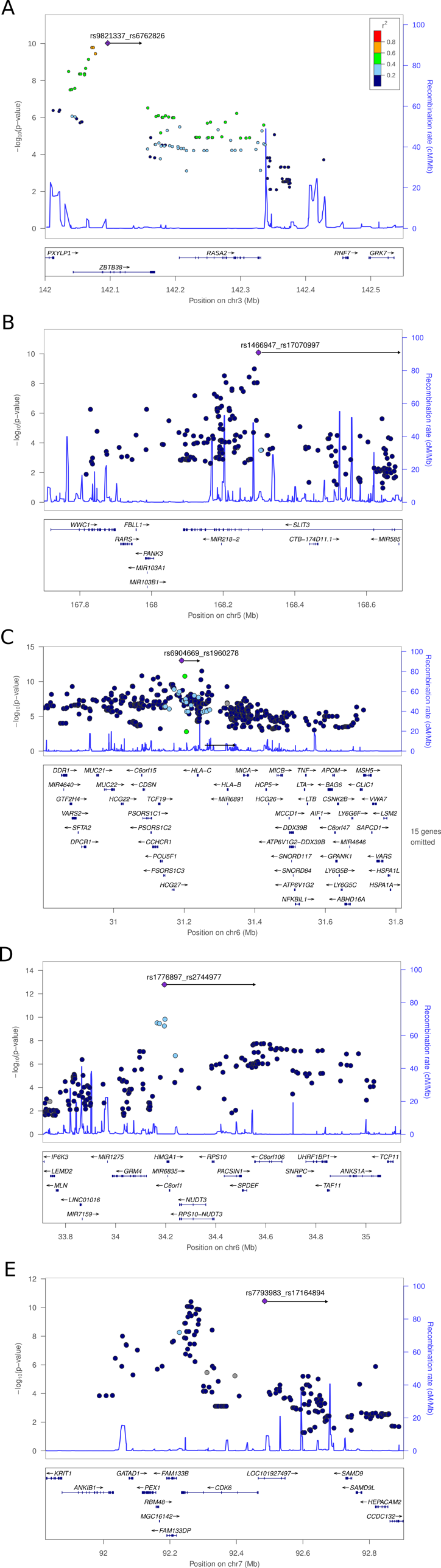

Supplement: Supplementary file 5 — Supplementary material 5 (DOCX 2170 kb) [file 439_2017_1842_MOESM5_ESM.docx]
