# Supplementary material for: Genome-wide compound heterozygote analysis highlights alleles associated with adult height in Europeans
Source: Hum Genet. 2017 Sep 18;136(11):1407–17. doi: 10.1007/s00439-017-1842-3 (PMC5702380; doi:10.1007/s00439-017-1842-3)
Supplement: Supplementary file 6 — Supplementary material 6 (PDF 15 kb) [file 439_2017_1842_MOESM6_ESM.pdf]

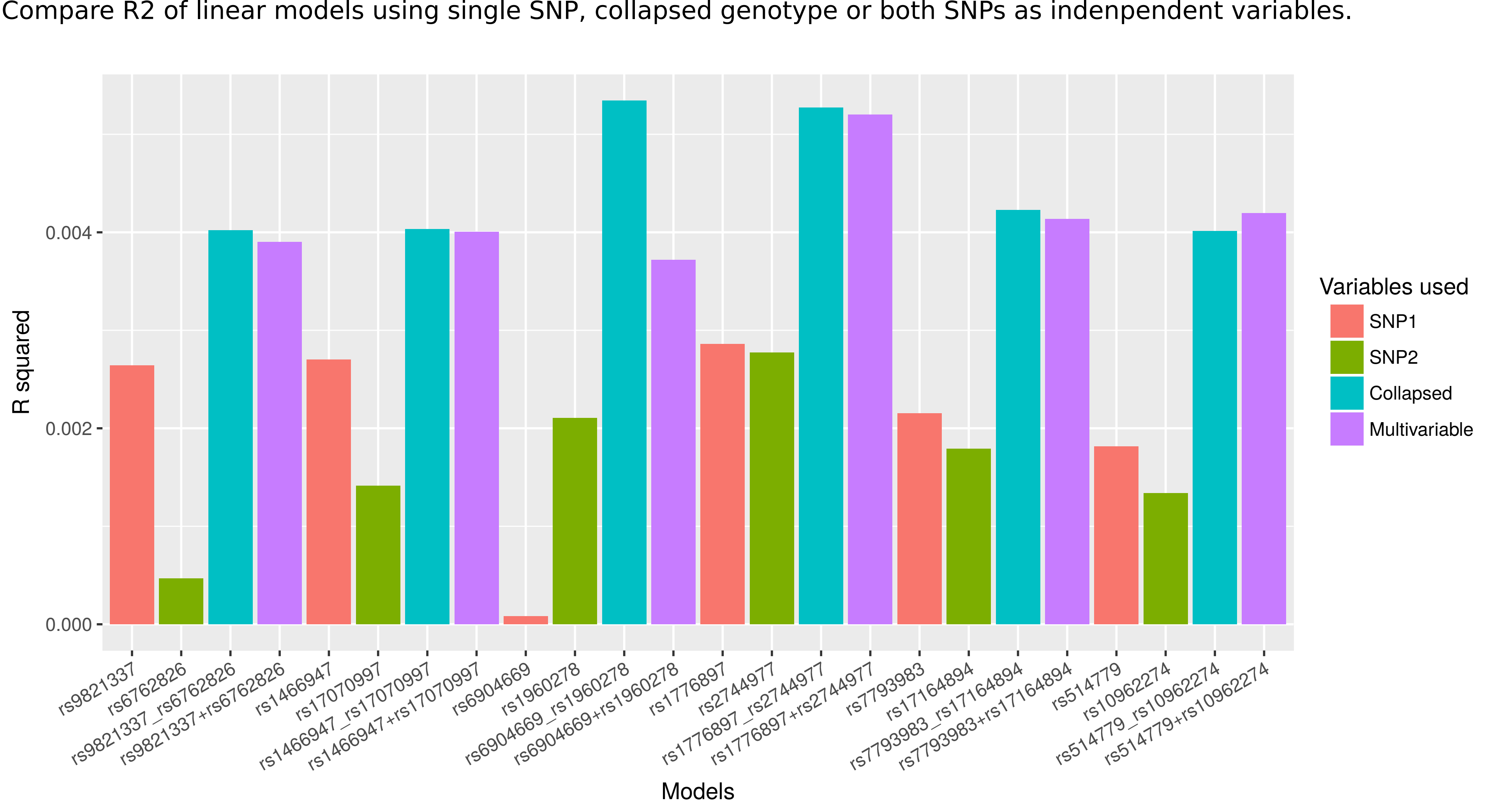
Compare R2 of linear models using single SNP, collapsed genotype or both SNPs as indenpendent variables.
R squared
0.000
0.002
0.004
Models
rs9821337
rs6762826
rs9821337_rs6762826
rs9821337+rs6762826
rs1466947
rs17070997
rs1466947_rs17070997
rs1466947+rs17070997
rs6904669
rs1960278
rs6904669_rs1960278
rs6904669+rs1960278
rs1776897
rs2744977
rs1776897_rs2744977
rs1776897+rs2744977
rs7793983
rs17164894
rs7793983_rs17164894
rs7793983+rs17164894
rs514779
rs10962274
rs514779_rs10962274
rs514779+rs10962274
Variables used
SNP1
SNP2
Collapsed
Multivariable
