# Supplementary material for: Genome-wide compound heterozygote analysis highlights alleles associated with adult height in Europeans
Source: Hum Genet. 2017 Sep 18;136(11):1407–17. doi: 10.1007/s00439-017-1842-3 (PMC5702380; doi:10.1007/s00439-017-1842-3)

Illustration of homozygosity, double heterozygosity and compound heterozygosity. (A) Same mutation at the same position on both homologous chromosomes (homozygote). (B) Two different mutations on the same chromosome (double heterozygote). (C) Two different mutations, one on each chromosome (compound heterozygote).


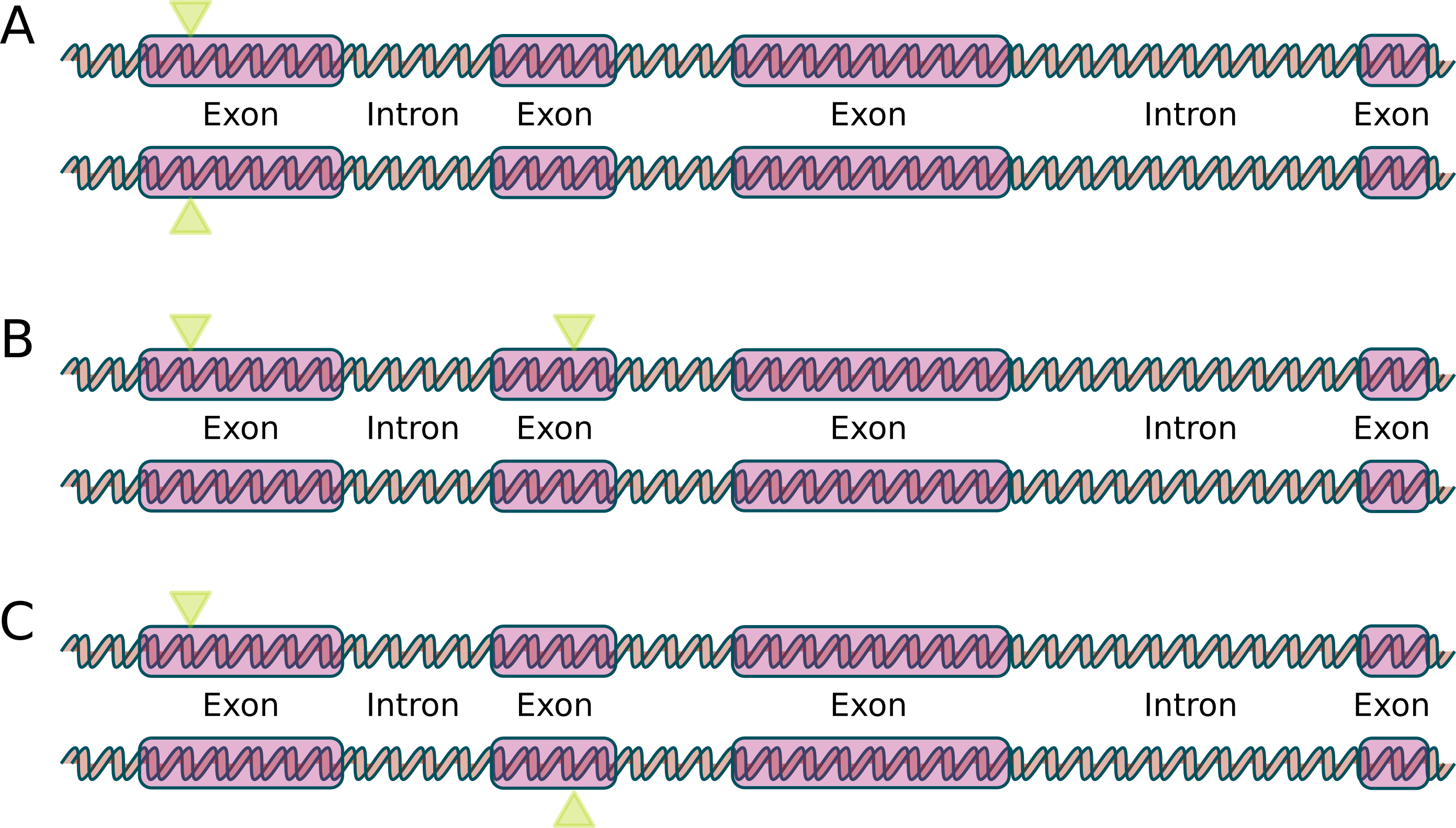

Supplement: Supplementary file 7 — Supplementary material 7 (DOCX 844 kb) [file 439_2017_1842_MOESM7_ESM.docx]
